# Incidental mass found on screening colonoscopy

**DOI:** 10.1093/jscr/rjag684

**Published:** 2026-08-11

**Authors:** Alan Amedi, Serhat Suzer, Jesse Zuckerman, Eiman Firoozmand

**Affiliations:** Department of Surgery, Cedars-Sinai Medical Center, Los Angeles, CA, United States; David Geffen School of Medicine at UCLA, Los Angeles, CA, United States; Department of Surgery, Cedars-Sinai Medical Center, Los Angeles, CA, United States; Department of Surgery, Cedars-Sinai Medical Center, Los Angeles, CA, United States

**Keywords:** intestinal tuberculosis, large bowel obstruction, colonoscopy

## Abstract

Intestinal tuberculosis accounts for 1%–3% of all tuberculosis cases worldwide and typically arises as a secondary manifestation of hematogenous spread rather than as a primary site of infection. The condition is characterized by an insidious onset, with patients often presenting late in the disease course with non-specific gastrointestinal symptoms. At advanced stages, clinical features may include signs of bowel obstruction or perforation, closely mimicking other conditions such as colorectal malignancy, colitis, or inflammatory bowel disease, posing a significant diagnostic challenge. We report a rare case of isolated intestinal tuberculosis presenting as a partial large bowel obstruction. The patient successfully underwent a right hemicolectomy followed by anti-tuberculosis pharmacotherapy, resulting in complete restoration of normal bowel function and eradication of tuberculosis.

## Introduction

An intestinal granuloma caused by tuberculosis is distinguishable from other granulomatous conditions by their histopathological features; typically consisting of multiple aggregates of confluent epithelioid histiocytes with multinucleated giant cells, often displaying caseating necrosis in response to *Mycobacterium tuberculosis* infection in the gastrointestinal tract [1, 2].

The cellular composition of a granuloma includes blood-derived infected and uninfected macrophages, foamy macrophages, epithelioid cells (uniquely differentiated macrophages), and multinucleated Langerhans giant cells, typically surrounded by a ring of lymphocytes. In intestinal tuberculosis specifically, ulcers may be lined by conglomerate epithelioid histiocytes, and there is often disproportionate submucosal inflammation [2]. The granuloma serves as a physiological barrier to contain the infection and concentrate the immune response, though it also limits antibiotic penetration and can harbor persistent bacteria.

The most commonly affected sites are the ileocecal valve (66%), terminal ileum (47%), and cecum (39%), where these granulomas form the pathological hallmark of intestinal tuberculosis [3]. In this case we present a patient with vague abdominal symptoms found to have a mass on screening colonoscopy causing partial obstruction of the colon discovered to be a granulomatous lesion concerning for tuberculosis.

## Case report

**Figure 1:**
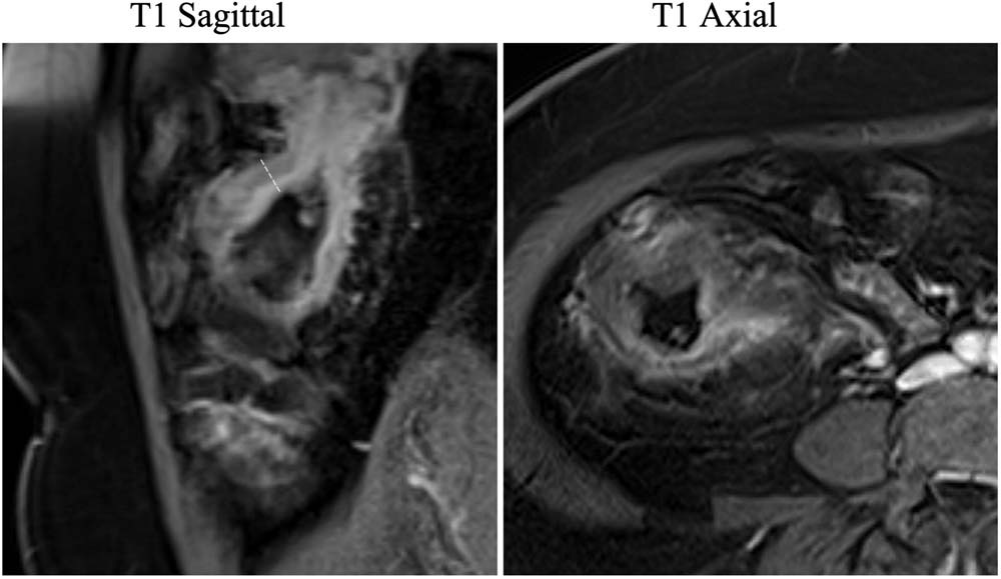
Pre-operative MRI of the abdomen and pelvis. There is an approximated 4–5 cm of decompressed colon/cecum with inner layer hyperenhancement and bowel wall thickening measuring up to 1.2 cm with involvement of the ileocecal junction and extension into the terminal ileum. There is no abnormal upstream dilation of the small bowel.

A 66-year-old Hispanic woman with a medical history of pulmonary nodules, and previous hysterectomy presented for screening colonoscopy, endorsing constipation with small caliber bowel movements along with tenesmus for multiple years. She denied hematochezia or melena, nausea, vomiting, fevers, night sweats, or weight loss. Her abdomen was soft and mildly tender in the right upper quadrant. She had no family history of cancer or inflammatory bowel disease (IBD). Her colonoscopy demonstrated a large stricturing lesion at the hepatic flexure which was not traversable by a standard colonoscope. Biopsies obtained during colonoscopy were negative for malignancy but showed evidence of inflammation, ulceration, and granulomas with negative acid-fast bacilli (AFB). Radiologic review noted that this was atypical for IBD (Fig. 1). Magnetic resonance imaging (MRI) was obtained showing hyperenhancement of the bowel wall consistent with severe focal colitis and an associated stricture. Further inflammatory work-up revealed a normal calprotectin level of 28, a normal carcinoembryonic antigen level of 2.3, and an elevated C reactive protein of 20.8. Due to the presence of her known pulmonary nodules, the patient underwent work-up for tuberculosis (TB). She had a positive QuantiFERON-TB gold test, which was followed by two negative sputum AFB tests, with negative bronchial lavage cultures and a negative transbronchial biopsy of the pulmonary nodules, ruling out TB.

**Figure 2:**
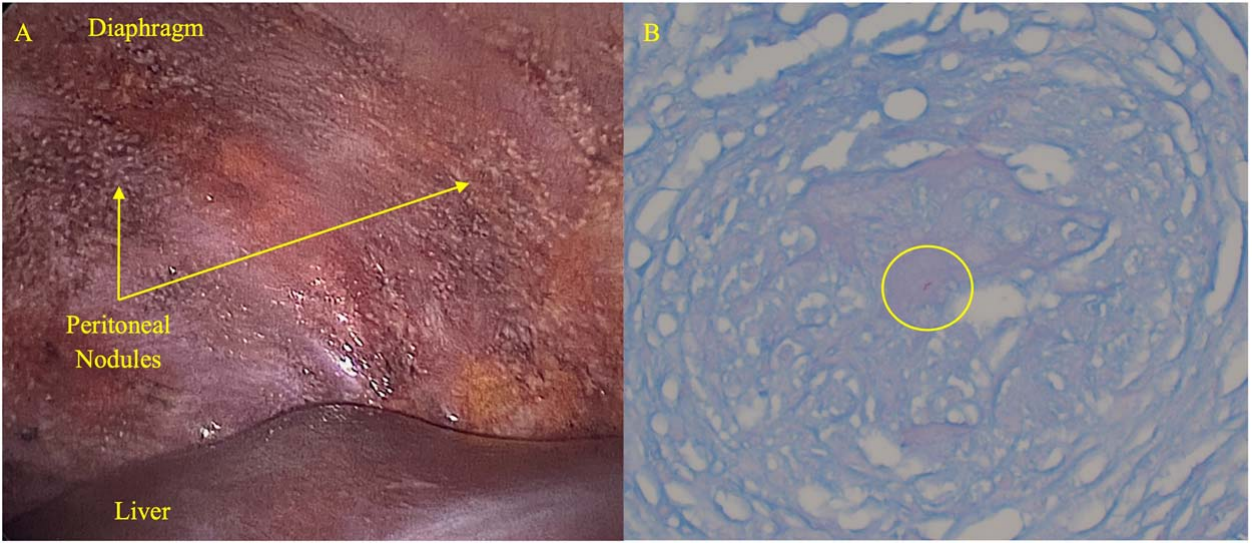
Intra-operative image of the peritoneal cavity and final pathology. (A) Once the abdominal cavity was entered laparoscopically, numerous lesions were identified covering the peritoneum. Nodules were excised and sent to pathology. (B) Final pathology with AFB staining revealing necrotizing granulomas and presence of *Mycobacterium tuberculosis*.

Upon entry into the abdomen, lesions were found covering the peritoneum concerning for malignancy (Fig. 2). The tattooed colon was identified at the hepatic flexure with a large mass adherent to the abdominal wall and associated extensive diaphragmatic nodularity. Intraoperative frozen biopsies of the peritoneal disease revealed granulomas with foci of necrosis but no malignancy—the decision was made to proceed with a right hemicolectomy with a side-to-side ileotransverse anastomosis. The post-operative course was unremarkable. Final pathology of the right colon demonstrated numerous granulomas with foci of necrosis in the colonic mucosa and AFB identified on staining of the lymph node. There were a total of six lymph nodes with granulomas present.

The patient’s diet was slowly advanced as bowel function returned. She was in isolation until TB was confirmed after which she was started on ethambutol, isoniazid, pyrazinamide, and rifampin. She was then discharged home with close follow-up and isolation precautions. Three months after her surgery, she endorsed no abdominal pain or distention with daily bowel movements. She continues to follow closely with the infectious disease team until completion of her anti-TB therapy after which follow up imaging will be done.

## Discussion

TB is still the world’s leading cause of death from infection as of 2023 with a reported 8.2 million people newly diagnosed [4]. Abdominal TB can be found in 1%–3% of global TB cases; however, the rate can vary depending on geographic region [5]. Abdominal TB is rarely primarily inoculated; more often spreading via a hematogenous route with subsequent reactivation or via lymphatics leading to miliary TB. Ingestion of bacilli from sputum or an alternative infected source can also lead to intestinal TB [6].

Intestinal TB has an indolent course with vague and mild symptoms. Presentation often occurs late in the disease course when the patient becomes symptomatic from obstruction or perforation, mimicking other disorders including malignancy, colitis or IBD, making clinical diagnosis difficult. In this case, the obstruction was incidentally found on screening for colon cancer. Interestingly, TB was initially ruled out due to lack of pulmonary involvement. Her sputum cultures and transbronchial biopsy were both negative for TB. However, there are varying reports of pulmonary involvement with intestinal TB ranging from 25%–91% [3, 6–8]. Intestinal TB without pulmonary involvement is possible. Uncomplicated cases of intestinal TB can be treated medically with a trial of anti-TB medication; however, resection is required in the setting of obstruction, perforation, or if the diagnosis remains in doubt [9].
